# RaggedExperiment: the missing link between genomic ranges and matrices in Bioconductor

**DOI:** 10.1093/bioinformatics/btad330

**Published:** 2023-05-19

**Authors:** Marcel Ramos, Martin Morgan, Ludwig Geistlinger, Vincent J Carey, Levi Waldron

**Affiliations:** Epidemiology and Biostatistics, Graduate School of Public Health and Health Policy, City University of New York, New York, NY 10027, United States; Institute for Implementation Science and Population Health, City University of New York, New York, NY 10027, United States; Biostatistics and Bioinformatics, Roswell Park Comprehensive Cancer Center, Buffalo, NY 14203, United States; Biostatistics and Bioinformatics, Roswell Park Comprehensive Cancer Center, Buffalo, NY 14203, United States; Epidemiology and Biostatistics, Graduate School of Public Health and Health Policy, City University of New York, New York, NY 10027, United States; Institute for Implementation Science and Population Health, City University of New York, New York, NY 10027, United States; Channing Division of Network Medicine, Brigham and Women’s Hospital and Harvard Medical School, Boston, MA 02115, United States; Epidemiology and Biostatistics, Graduate School of Public Health and Health Policy, City University of New York, New York, NY 10027, United States; Institute for Implementation Science and Population Health, City University of New York, New York, NY 10027, United States

## Abstract

**Summary:**

The RaggedExperiment R / Bioconductor package provides lossless representation of disparate genomic ranges across multiple specimens or cells, in conjunction with efficient and flexible calculations of rectangular-shaped summaries for downstream analysis. Applications include statistical analysis of somatic mutations, copy number, methylation, and open chromatin data. RaggedExperiment is compatible with multimodal data analysis as a component of MultiAssayExperiment data objects, and simplifies data representation and transformation for software developers and analysts.

**Motivation and Results:**

Measurement of copy number, mutation, single nucleotide polymorphism, and other genomic attributes that may be stored as VCF files produce “ragged” genomic ranges data: i.e. across different genomic coordinates in each sample. Ragged data are not rectangular or matrix-like, presenting informatics challenges for downstream statistical analyses. We present the RaggedExperiment R/Bioconductor data structure for lossless representation of ragged genomic data, with associated reshaping tools for flexible and efficient calculation of tabular representations to support a wide range of downstream statistical analyses. We demonstrate its applicability to copy number and somatic mutation data across 33 TCGA cancer datasets.

## 1 Introduction

“Ragged” genomic ranges data arise from the disparate measurements per assayed specimen, such as from the analysis of DNA copy number, somatic mutation, methylation, open chromatin, single nucleotide polymorphisms, and other genomic coordinate analyses. Ragged data can be represented efficiently on disk using Variant Call Format (VCF) (Danecek *et al.* 2011). In Bioconductor, the GenomicRanges software infrastructure which includes GRanges and GRangesList, provides a general framework for the representation of genomic coordinates (Lawrence *et al.* 2013). GRanges represents an individual sample whereas GRangesList is the list-like extension for multiple samples. However, many downstream statistical and visualization analyses require a matrix-like dataset as input. While often necessary, such summarization can be complex to perform, lossy, and can greatly expand the dataset memory footprint, and is therefore best performed at a late step and on an as-needed basis. There has been no Bioconductor data class for lossless representation of ragged genomic data within the MultiAssayExperiment (Ramos *et al.* 2017) ecosystem of packages for multi’omic data analysis, or to facilitate flexible conversion to matrix-like representations such as number of coding mutations or segmented copy number per gene.

We therefore present the RaggedExperiment Bioconductor/R package for representing ragged genomic ranges from multiple samples, and to provide flexible and efficient tools for matrix-format summarization across identical ranges in each sample. These tools include computation on predetermined ranges such as genes, on overlapping genomic ranges across multiple samples, on unique ranges, and on replicated ranges. RaggedExperiment efficiently represents mutation and copy number data across The Cancer Genome Atlas (TCGA) and cBioPortal (Gao *et al.* 2013), and enables a wide range of downstream analyses.

## 2 Materials and methods

Internally, the RaggedExperiment class is an S4 class extending the Annotated base class, with a slot “assays” containing a GRangesList class object as defined by the GenomicRanges Bioconductor package (Lawrence *et al.* 2013), and additional slots “rowidx” and “colidx,” containing integer row and column indices. The external user interface RaggedExperiment mimics where possible the RangedSummarizedExperiment class (Huber *et al.* 2015). Methods for data representation and management include:

1. a function RaggedExperiment for construction from GRanges or GRangesList objects and optional phenotype/ranges/metadata as listed below
2. colData for getting/setting sample phenotype data
3. rowRanges getting/setting genomic coordinates
4. square-bracket [i, j] subsetting along *rowidx* (elements of genomic ranges) and *colidx* (samples) and returning another RaggedExperiment
5. overlapsAny and subsetByOverlaps for respectively identifying (and returning a logical vector) or subsetting (and returning a RaggedExperiment) by overlaps with a query vector of genomic ranges
6. assay, assays, and assayNames for getting/setting ranges data comparably to SummarizedExperiment
7. seqinfo for getting/setting chromosome sequence naming conventions (based on the GenomeInfoDb package)
8. mcols for getting/setting range metadata (comparable to GRanges and GRangesList objects)
9. dim, dimnames, length, and show functions
10. coercion methods to and from GRangesList

Finally, RaggedExperiment provides the sparseAssay, compactAssay, disjoinAssay, and qreduceAssay functions for different types of conversion to matrix format, as described in the Results. These functions employ computationally efficient range algebra from the GenomicRanges package. RaggedExperiment employs open development and issue tracking on GitHub, and distribution through bioconductor.org (Morgan and Ramos).

## 3 Results

**Figure 1.**
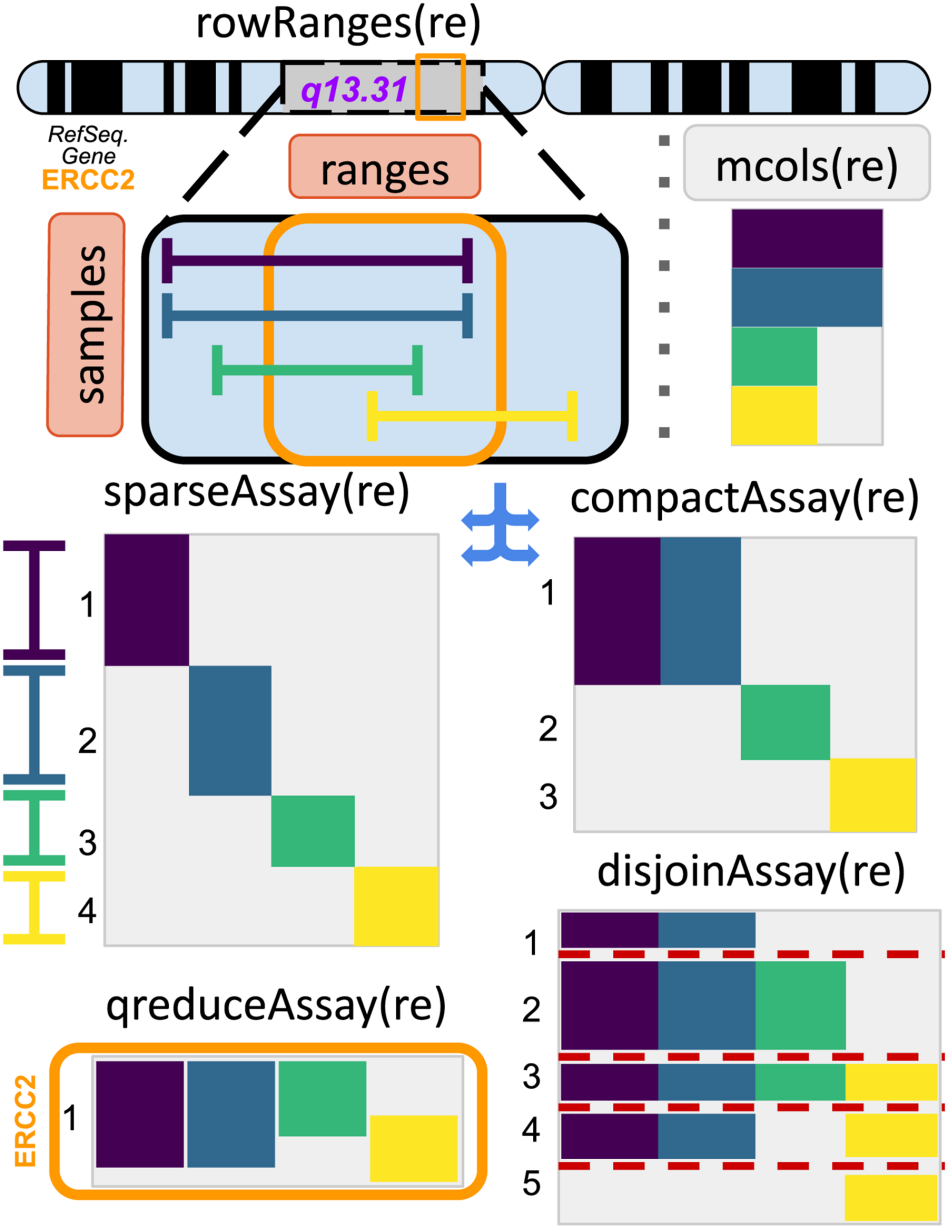
Schematic of the RaggedExperiment class and matrix conversion methods. The RaggedExperiment object provides representation of irregular range measurements on a set of samples (top; each whisker represents a genomic range from a single sample and overlaps can be seen down the stack of ranges. Top right; range metadata are stored internally; accessible with mcols). sparseAssay provides a fast rectangular and sparse representation with always one row per range; compactAssay combines identically overlapping ranges; disjoinAssay disjoins all overlapping regions across the data, and qreduceAssay summarizes observations and measurements to user-specified genomic regions of interest, e.g. within the ERCC2 gene. Solid colored blocks represent numeric or even character type data obtained from mcols. Matrices show row numbers.

Figure 1 shows a schematic representation of the four general approaches to combining and reshaping ranged coordinates in a RaggedExperiment representation (labeled “re”). At the top, we represent the row ranges component of the RaggedExperiment as a set of samples with dissimilar range measurements given by the height of the rectangles. The four different transformations described below cover broad and flexible use cases for analysis and higher-level package development.

### 3.1 sparseAssay: maintaining all ranges

sparseAssay is the most straightforward conversion: the resulting matrix has one row per input genomic range observations across all samples, and one column for each individual sample, even if samples share identical genomic coordinates. Since this function produces very sparse matrices (most values are missing), we have added an option for memory-efficient sparseMatrix representation from the Matrix package (Bates *et al.* 2010). It is the fastest way to convert data from a nested GRangesList structure to a rectangular sparse matrix.

### 3.2 compactAssay: combine identical ranges

compactAssay provides a slightly more dense matrix representation compared to sparseAssay, finding and combining identical ranges. It differs from sparseAssay only if there are identical input ranges, as these are included in the same row of the output matrix. compactAssay can be used, e.g. to convert open chromatin regions, where many overlap across biological cells, to a regions x cells matrix with overlapping regions merged to the same row. It is also useful for converting Single Nucleotide Polymorphism (SNP) data from a VCF file type format to a SNP x samples matrix. A sparseMatrix representation is also available.

### 3.3 disjoinAssay: disjoin overlapping ranges

Disjoint ranges are a set of ranges with no overlap. The disjoin procedure creates ranges from a union of endpoints obtained from a set of genomic ranges, by applying the disjoin operation across all samples to fragment all ranges in the data. Users can provide a function (e.g. mean) to combine overlapping ranges after the fragmentation. Non-disjoint ranges are not collapsed. disjoinAssay can be used, e.g. to fragment partially overlapping segmented copy number data and identify regions of frequent alteration across samples (da Silva *et al.* 2020).

### 3.4 qreduceAssay: summarize across specified ranges of interest

qreduceAssay summarizes metadata across pre-specified genomic regions of interest, such as genes, and is the most important reduce function for many use cases. The user provides query ranges with which to summarize regions across all samples, and a function to produce output matrix values from input metadata. This user-provided function must have three arguments to define rules for iteration and summarization over regions of interest. We provide documentation(Morgan and Ramos) and convenience code (Ramos *et al.* 2019) for summarizing TCGA somatic mutation data as a matrix of zeros and ones, with one row per protein-coding gene and one column per patient, where any gene containing one or more of any kind of non-silent mutation is coded as “1” and genes with silent or no mutations mutations are coded as “0.” Such rules can be extended to include flanking regions, to count total numbers of mutations, etc.

### 3.5 Benchmarking

RaggedExperiment fills a gap in providing efficient, flexible conversion between “ragged” genomic data and matrix format for which we are not aware of a direct analogy to benchmark against. The most commonly used alternative is to store and distribute a pre-computed matrix of data on fixed genomic features such as regions of recurrent copy number, genes containing mutations, or SNPs, across every sample. To demonstrate the advantage of the RaggedExperiment lossless representation of VCF-like data and efficient reduction options, we used RaggedExperiment to represent mutation and copy number data from the Breast Invasive Carcinoma (BRCA) cancer type in TCGA, through the curatedTCGAData package (Ramos *et al.* 2020b). Table 1 shows a memory footprint size comparison between RaggedExperiment, sparse Matrix (Bates *et al.* 2010), and the native R matrix representations for CNA-seq and mutation data. Segmented copy number alterations (CNA) from the Illumina HiSeq sequencing platform consume a total of 0.2 MB as RaggedExperiment, comparable to sparse Matrix’s 0.3 MB, and 1 MB using the traditional matrix. BRCA somatic mutation data consumes about 71 MB as RaggedExperiment compared to 680 MB when using a traditional character matrix, representing a nearly 10-fold decrease in memory footprint. The Matrix data representation does not support character-type data values.

**Table 1.** Comparison of memory footprints reported by object.size() between RaggedExperiment and alternatives.

Assay^a^	Data type	RaggedExperiment	sparse Matrix	matrix (reduced rows)	matrix (sparse)
CNASeq	Numeric	0.2	0.3	1	1.9
CNASeq^b^	Numeric	0.2	0.3	0.9	1.7
Mutation	Character	70.6	–	680.3	726.2
Mutation^b^	Character	37.6	–	351.3	375.5

a All units are in megabytes (MB). Data described are from the BRCA TCGA dataset.

b Assay features are limited to genic regions.

To demonstrate efficiency in conversion, we used qReduceAssay to convert the largest of these RaggedExperiment objects, segmented copy number variation data for 284 458 ranges on 2199 BRCA samples, to a matrix of numeric copy number on 22 917 protein-coding genes across the same 2199 samples. This operation converted an 8.6 MB RaggedExperiment object to a 407 MB SummarizedExperiment object containing a matrix assay of dimensions 22 917 × 2199, in ∼2 min on a single CPU. Similar operations on smaller TCGA datasets of several hundred specimens across all human genes completed in less than a minute. Code for these computations is available on GitHub (https://github.com/waldronlab/RaggedExperiment_SoftNote).

## 4 Discussion

RaggedExperiment fills a need among core Bioconductor data classes for lossless representation of disparate ranged measurements on a set of samples, with flexible and efficient calculation of matrix-like representations for statistical analysis and compatibility with the MultiAssayExperiment (Ramos *et al.* 2017) data class for multi’omic experiments. Several Bioconductor packages have already adopted RaggedExperiment for multi’omic analysis, demonstrating breadth of application for genomic analysis: CNVRanger (da Silva *et al.* 2020) for population copy number and eQTL analysis, omicsPrint (van Iterson *et al.* 2018) for multi’omic visualization, maftools (Mayakonda *et al.* 2018) for analysis of somatic variants in cancer, RTCGAToolbox (Samur 2014) for representing TCGA data, and MultiDataSet (Hernandez-Ferrer *et al.* 2017) for multi’omic analysis. RaggedExperiment is applicable to bulk or single-cell data and to any species, as demonstrated by its use in the SingleCellMultiModal data package (Ramos *et al.* 2020a) for analysis and re-distribution of single-cell DNA copy number from embryonic mouse cells by the G&T-seq assay (Macaulay *et al.* 2015).

## 5 Conclusions

The RaggedExperiment package fills a need for lossless representation of disparate genomic ranges across multiple cells or specimens, coupled with efficient and flexible calculation of matrix-like summaries for downstream analysis. RaggedExperiment is applicable to any species, to any assay generating data on genomic ranges (such as somatic mutations, copy number, methylation, and open chromatin), and to general statistical analysis, e.g. identifying differentially altered genomic regions across two experimental conditions. RaggedExperiment simplifies such analyses for both data analysts and other software developers, and will receive long-term maintenance as a “core” Bioconductor data class.

## Data Availability

RaggedExperiment is publicly available under an Artistic 2.0 license at Bioconductor (https://dx.doi.org/doi:10.18129/B9.bioc.RaggedExperiment) with open development and issue tracking on GitHub (https://github.com/Bioconductor/RaggedExperiment).

## References

[btad330-B1] Bates D, Maechler M, Jagan M et al *Matrix: Sparse and Dense Matrix Classes and Methods*. R package version 1.5-4.1.

[btad330-B2] Danecek P , Auton A, Abecasis G et al; 1000 Genomes Project Analysis Group. The variant call format and VCFtools. Bioinformatics 2011;27:2156–8. 21653522 10.1093/bioinformatics/btr330 PMC3137218

[btad330-B3] Gao J , Aksoy BA, Dogrusoz U et al Integrative analysis of complex cancer genomics and clinical profiles using the cBioPortal. Sci Signal 2013;6:l1. 10.1126/scisignal.2004088 PMC4160307 23550210

[btad330-B4] Hernandez-Ferrer C , Ruiz-Arenas C, Beltran-Gomila A et al MultiDataSet: an R package for encapsulating multiple data sets with application to omic data integration. BMC Bioinformatics 2017;18:36. 28095799 10.1186/s12859-016-1455-1 PMC5240259

[btad330-B5] Huber W , Carey VJ, Gentleman R et al Orchestrating high-throughput genomic analysis with bioconductor. Nat Methods 2015;12:115–21. 25633503 10.1038/nmeth.3252 PMC4509590

[btad330-B6] van Iterson M , Cats D, Hop P et al; BIOS Consortium. omicsPrint: detection of data linkage errors in multiple omics studies. Bioinformatics 2018;34:2142–3. 29420690 10.1093/bioinformatics/bty062

[btad330-B7] Lawrence M , Huber W, Pagès H et al Software for computing and annotating genomic ranges. PLoS Comput Biol 2013;9:e1003118. 23950696 10.1371/journal.pcbi.1003118 PMC3738458

[btad330-B8] Macaulay IC , Haerty W, Kumar P et al G&T-seq: parallel sequencing of single-cell genomes and transcriptomes. Nat Methods 2015;12:519–22. 25915121 10.1038/nmeth.3370

[btad330-B9] Mayakonda A , Lin D-C, Assenov Y et al Maftools: efficient and comprehensive analysis of somatic variants in cancer. Genome Res 2018;28:1747–56. 30341162 10.1101/gr.239244.118 PMC6211645

[btad330-B10] Morgan M , Ramos M. *RaggedExperiment*. Bioconductor package version 1.24.0.

[btad330-B11] Ramos M , Eckenrode K, Waldron L et al *SingleCellMultiModal. Bioconductor package version 1.12.2.*.

[btad330-B12] Ramos M , Geistlinger L, Oh S et al Multiomic integration of public oncology databases in bioconductor. JCO Clin Cancer Inform2020b;4:958–71. 33119407 10.1200/CCI.19.00119 PMC7608653

[btad330-B13] Ramos M , Schiffer L, Re A et al Software for the integration of multiomics experiments in bioconductor. Cancer Res 2017;77:e39–42. 29092936 10.1158/0008-5472.CAN-17-0344 PMC5679241

[btad330-B14] Ramos M, Schiffer L, Waldron L. *TCGAutils: TCGA Utility Functions for Data Management.* Bioconductor package version 1.20.2.

[btad330-B15] Samur MK. RTCGAToolbox: a new tool for exporting TCGA firehose data. PLoS One 2014;9:e106397. 25181531 10.1371/journal.pone.0106397 PMC4152273

[btad330-B16] da Silva V , Ramos M, Groenen M et al CNVRanger: association analysis of CNVs with gene expression and quantitative phenotypes. Bioinformatics 2020;36:972–3. 31392308 10.1093/bioinformatics/btz632 PMC9887538

